# Breastfeeding vs. Formula Feeding and Maternal Sexuality among Polish Women: A Preliminary Report

**DOI:** 10.3390/healthcare12010038

**Published:** 2023-12-23

**Authors:** Anna Weronika Szablewska, Anna Michalik, Agnieszka Czerwińska-Osipiak, Sebastian Artur Zdończyk, Marcin Śniadecki, Katarzyna Bukato, Wanda Kwiatkowska

**Affiliations:** 1 Department of Obstetrical and Gynecological Nursing, Institute of Nursing and Midwifery, Faculty of Health Sciences with the Institute of Maritime and Tropical Medicine, Medical University of Gdansk, Debinki 7, 80-211 Gdansk, Poland; anna.michalik@gumed.edu.pl (A.M.); agnieszka.czerwinska-osipiak@gumed.edu.pl (A.C.-O.); wkwiatkowska@gumed.edu.pl (W.K.); 2 Department of General Pedagogy and Fundamentals of Education, Institute of Pedagogy, Pomeranian University in Słupsk, Arciszewskiego 22a, 76-200 Słupsk, Poland; sebastian.zdonczyk@apsl.edu.pl; 3 Department of Hematology and Bone Marrow Transplantation, Provincial Specialist Hospital Named after J. Korczak in Słupsk sp. z o.o., Hubalczyków 1, 76-200 Słupsk, Poland; 4 Department of Gynecology, Gynecologic Endocrinology and Gynecologic Oncology, Medical University of Gdańsk, Marian Smoluchowski 17, 80-214 Gdańsk, Poland; marcin.sniadecki@gumed.edu.pl (M.Ś.); kasiabukato@gumed.edu.pl (K.B.)

**Keywords:** breastfeeding, sexual functioning, postpartum period

## Abstract

Although postpartum sexual problems are common, there is a poor understanding of the underlying influencing factors and the impact of the infant feeding method on the mother’s sexual life. A cross-sectional control study was conducted with a group of 253 women during their postpartum period. This study aimed to investigate the effects of different infant feeding methods on female sexual life after childbirth. The study followed the STROBE guidelines for cross-sectional control analysis. The study design included a questionnaire characterizing sociodemographic, obstetric and breastfeeding variables and the PL-FSFI (Female Sexual Function Index). The authors collected the data in compliance with the CAWI (Computer-Assisted Web Interview) research methodology—an interview conducted via an Internet channel. Each respondent received and completed the survey provided to them via the same online link. This study included women in the postpartum period: 170 breastfeeding women (study group) and 83 formula-feeding women (control group). There were statistically significant difference between the groups that practiced different types of breastfeeding. Out of all the PL-FSFI-assessing domains, the highest average score for the whole group correlated with satisfaction and the lowest score correlated with lubrication use. Our findings indicate that women practicing only breastfeeding are more likely to develop sexual problems. In order to maintain sexual health and promote long-term breastfeeding, extensive and professional counseling is needed for couples about postpartum sexuality and the factors that affect it, such as breastfeeding.

## 1. Introduction

Global health organizations, such as the World Health Organization (WHO), United Nations International Children’s Emergency Fund (UNICEF), and the American Academy of Pediatrics (AAP), advocate for exclusive breastfeeding during the first six months of an infant’s life, with continued breastfeeding recommended for up to two years of age or beyond for optimal growth [1,2,3,4]. Childbirth itself initiates a cascade of changes in a woman’s life. New mothers grapple with a myriad of factors impacting intimate and sexual relationships, such as role incompatibility, extreme fatigue, sleep deprivation, and concerns about evolving body image, all leading to changes in libido [5,6,7,8,9,10,11]. Despite considerable benefits of breastfeeding, it can introduce challenges to a woman’s sexuality and affect the quality of life in this area [12]. While existing research primarily focuses on sexual functioning during pregnancy or after childbirth, the relationship between infant feeding methods and sexuality remains understudied and less well understood.

The postpartum period witnesses a gradual resumption of sexual activity, with 80–93% of new mothers engaging in intercourse within three months of delivery [13]. However, this period is also marked by challenges, as two out of three women encounter difficulties related to sexual functioning, including decreased libido, difficulty achieving orgasm, vaginal dryness, and dyspareunia [14]. Factors contributing to reduced postnatal sexual activity include diminished interest in sex, postpartum pain, tender breasts, and lactation-related concerns (for example, uncontrolled milk leakage may be the reason for avoiding sexual intercourse for fear of the partner’s reactions) [5,15,16,17,18]. Notably, breastfeeding women report sexual dysfunction or inactivity more frequently than their non-breastfeeding counterparts [9]. Hormonal fluctuations during breastfeeding, particularly involving prolactin and androgen levels, contribute to the suppression of libido and interference with sexual response phases [10]. This is a physiological adaptation that aims to prioritize the nourishment of the infant and the demands of breastfeeding over sexual responsiveness [19]. Moreover, some studies suggest that the negative effect of breastfeeding on sexual function may be due to a higher level of prolactin and decreased levels of estrogen and progesterone, leading to vaginal dryness and atrophy, causing dyspareunia [12,20,21]. On the other hand, breastfeeding is associated with a reduction in perceived stress and negativity in mothers, due to the increased production of oxytocin, which is known as the “love and happiness hormone”, and an increased release of endorphins [22]. In addition androgens, including testosterone, contribute to sexual desire and arousal in both men and women. During pregnancy and the postpartum period, there can be a temporary decrease in androgen levels, particularly testosterone. This hormonal fluctuation can lead to changes in sexual responsiveness, potentially contributing to decreased libido and other sexual function issues [23].

Understanding the interplay between these hormonal changes and sexual functioning is crucial in comprehending the challenges that some women may face during the postpartum period. Female sexual dysfunction (FSD) emerges as a common yet underestimated problem with profound effects on women’s quality of life [11]. Importantly, this phenomenon remains understudied, particularly in the context of Polish women during the postpartum period.

Recent data show that 43% of women complain of at least one sexual problem [24,25]. The most recent Polish data on the prevalence of sexual dysfunction comes from 2005 and indicates that 25% of women experienced reduced interest in sexual activity and disorders of desire, 17% of women reported difficulties in reaching an orgasm, and 8% suffered from its absence. One of the most common sexual disorders in women is sexual interest disorder (also known as arousal disorder). The same study also showed that 13% of women are unable to have sexual intercourse due to dyspareunia and 2% due to vaginismus [26]. After childbirth, a woman transitions from partner, to mother and parent, while the couple transitions to family. There are new factors in the postpartum period, including household changes and new responsibilities. Breastfeeding is an essential element. It can have a positive effect on maternal sexual health thanks to oxytocin levels. However, it can also affect adversely, bringing about reduced sexual desire and associated lower sexual satisfaction for the woman and her partner [27].

Our study breaks new ground by addressing this under-researched intersection between infant feeding methods and women’s postpartum sexual health. While the conference paper ‘Female sexual functioning and level of self-esteem after childbirth–An introductory report’ [28] provided valuable preliminary insights, our current study builds upon this foundation by conducting a comprehensive cross-sectional analysis, utilizing additional measures. Our investigation further explores factors influencing sexual functioning after childbirth, particularly focusing on the type of infant feeding, thereby offering a more nuanced understanding of the dynamics at play in women’s lives during this crucial period. By examining the impact of different feeding practices on the sexual lives of postpartum women, our cross-sectional case–control study not only reveals significant differences between breastfeeding and formula-feeding groups, but also highlights the prevalence of specific dysfunctions such as lubrication problems and lack of sexual desire. Our innovative approach brings valuable insights, highlighting the urgent need for comprehensive and professional counselling for women and couples to improve postpartum sexual health and encourage long-term breastfeeding.

The main objective of this study was to analyze the impact of different infant feeding methods on the postpartum sexual functioning of Polish women, providing valuable information on the interaction between feeding practices and postpartum sexual health.

## 2. Materials and Methods

### 2.1. Study Design

This study was a cross-sectional control study to obtain objective and subjective data about sexual functioning after childbirth in groups of mothers who breastfeed and formula-feed their infants The study design included a questionnaire for characterizing sociodemographic, obstetric and breastfeeding variables and the PL-FSFI (Female Sexual Function Index) [29]. The Female Sexual Function Index is a questionnaire designed for women [24]. It consists of 19 items and aims to provide a multifaceted self-assessment of sexual functioning over the past four weeks. This tool allows for differentiation of sexual dysfunctions in the areas of desire, arousal, reaching orgasm, as well as feelings of sexual satisfaction. The scale is available in numerous language versions, whose psychometric and linguistic properties have been verified through numerous empirical studies. The high reliability of the presented tool can be identified as one of the reasons for its frequent use in research on female sexuality around the world. These studies involve very diverse groups of women. Points are assigned for each answer. The sum of the scores for the domain is multiplied by the domain factor. All domains are added up, and the total score may vary from 2.0 to 36.0 points. Female sexual dysfunction (FSD) includes disorders of desire/libido, arousal, pain or discomfort, and inhibited orgasm [30].

FSD (female sexual dysfunction for the Polish population) was identified if the total score was ≤27.5 points and below 3.9 for individual subscales. The Cronbach coefficient for this tool is 0.97 [19].

The study was conducted in accordance with the Declaration of Helsinki, and approved by the Institutional Review Board, i.e., the Independent Bioethics Committee for Scientific Research at the Medical University of Gdańsk, Poland (NKBBN/179/2015) for studies involving humans.

### 2.2. Participatnts and Settings

This study included 253 Polish women in the postpartum period:

- 170 breastfeeding women (study group);
- 83 formula-feeding women (control group).

**Figure 1:**
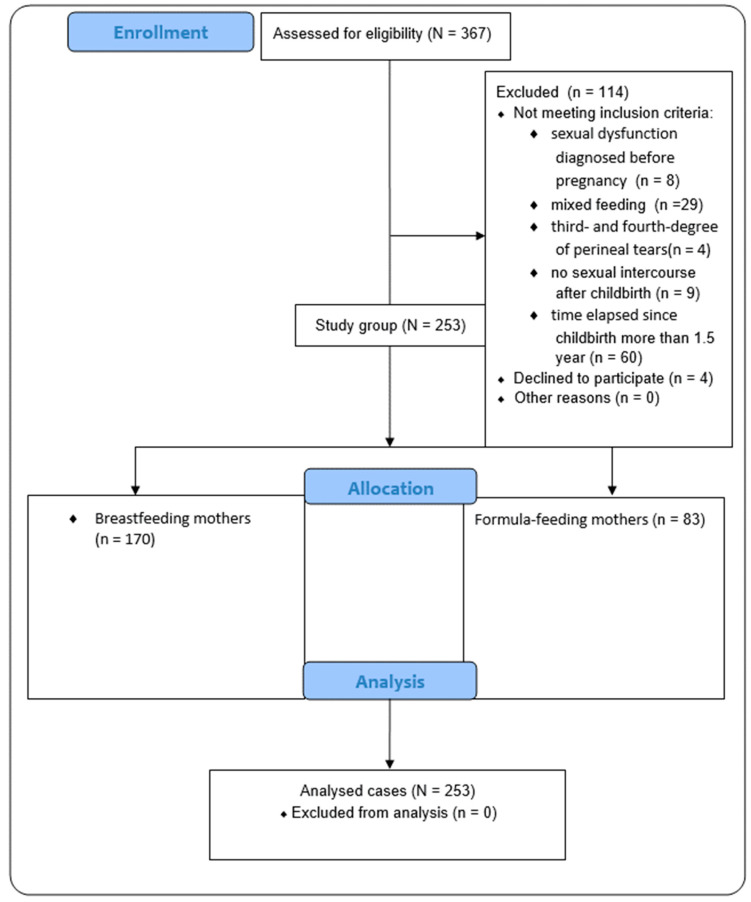
The flow of participants through the study.

The flow of participants through the study is presented on the Figure 1. 

All participants were informed of the study objectives and provided consent to respond to the questionnaire. The data collection period and respondents’ eligibility was 2015–2016. The authors collected the data in compliance with the CAWI (Computer-Assisted Web Interview) research methodology—an interview conducted via an Internet-based channel.

Each respondent received and completed the survey provided to them via the same online link. The eligibility screen was an integral part of the questionnaire. The questions were posted on social media accounts and groups dedicated to motherhood and breastfeeding. The survey contained social, demographic, and medical data (also connected with sexual behavior).

The context of the survey and the way it was conducted warrants classifying the sampling method as non-probability independent sampling, meaning that every participant in the target population has an equal chance of being included in the survey. In the case of surveys conducted via the Internet (CAWI), accessibility is wide-ranging, which promotes the diversity of participants. Sample independence is ensured because each participant is independent of other respondents, meaning that the result of one participant does not affect the results of the others. In the case of online surveys, where participants are not physically present in one place, their responses are independent of each other [31]. In a study of intimate issues such as sexual functioning, it is a crucial factor and a desirable aspect.

The procedures for recruiting participants included posting an online questionnaire on closed online groups dedicated to postpartum mothers (verification of their suitability was completed through the group administrators). Through active participation in these groups, we ensured access to a diverse group of women who were exclusively breastfeeding or feeding their infants with formula. Women were able to participate voluntarily and their access to the survey was provided through a single link published on the online platform. Access to the actual questionnaire was only granted to those whose initial responses matched the inclusion criteria. Providing a negative answer to any of the exclusion criteria resulted in termination of participation in the study. Data quality was controlled using various precautions such as avoiding multiple responses from the same person through unique identifiers or mechanisms to check the uniqueness of participation. In addition, the CAWI system allowed us to monitor the time spent completing the survey, which helped to identify any anomalies. All these steps ensured the reliability and quality of the data collected.

### 2.3. Exclusion Criteria

We excluded women with any sexual dysfunction and/or disorders diagnosed before pregnancy. We also excluded women using mixed methods of feeding newborns because we strived to focus on the differences between breastfeeding women (also continuing breastfeeding after including other foods in the diet) and women who did not declare breastfeeding at the time of the study (including not breastfeeding in the four weeks evaluated in the FSFI protocol). The next criterion included third- and fourth-degree perineal tears, which can affect the assessment of sexual functioning in each domain. We also excluded women who did not resume intercourse after childbirth (it can be due to medical conditions or the short time elapsed since childbirth) and women more than one and half years after labor because of a relatively small number of women who breastfeed for that amount of time in Poland [32]. 

### 2.4. Inclusion Criteria

We included women up to 18 months postpartum (regardless of the mode of completion of pregnancy) in the study. The American Academy of Pediatrics and the World Health Organization recommend exclusive breastfeeding for the first six months, with continued breastfeeding with gradually introduced appropriate complementary foods for up to two years of age or longer. We note that this time frame is adequate for both studied variables: breastfeeding (according to the recommendations) and resuming intercourse. 

### 2.5. Study Size and Statistical Methods

As this study aimed to obtain objective and subjective data using a cross-sectional control study design and questionnaires in its protocol, an estimated sample size at this stage was deemed sufficient. However, we checked whether the data used would be sufficient a priori using the G*power 3.1. (correlations: two independent Pearson, effect size q = 0.5; α err prob = 0.05; power = 0.95; allocation ratio N2/N1 = 2; result: sample size group 1 = 68; sample size group 2 = 135; total sample size = 203, using variable: breastfeeding mothers vs. formula feeding mothers). The calculated sample size was smaller than our group, and gaining greater access to patient data was possible.

Quantitative variables were characterized by arithmetic mean, standard deviation (SD), median, minimum and maximum value (range), and 95%CI (confidence interval) (domain scoring of PL-FSFI). Qualitative-type variables, on the other hand, were presented as counts and percentages. The Shapiro–Wilk test was used to test whether a quantitative variable came from a population with a normal distribution. The Leven (Brown–Forsythe) test was used to test the hypothesis of equal variances. 

Chi-square tests of independence were used for qualitative variables (using Yates correction for cell counts below 10, checking Cochran’s conditions, Fisher’s exact test) to assess the difference in the mean total score of PL-FSFI breastfeeding and formula-feeding groups and score in each domain score vs. type of feeding infant, time elapsed since childbirth, mode of birth, differences in persons who initiated sexual intercourse more often before pregnancy, in the last 4 weeks, and educational level. Logistic regression is used to obtain the odds ratio in the presence of more than one explanatory variable (domains: arousal, lubrication, orgasm, satisfaction vs. education level and time elapsed since childbirth and breastfeeding/formula feeding). All statistical analyses were performed using the Statistica 12.0 program and PQStat Software (2023). PQStat v.1.8.6.116. The level of significance was *p* < 0.05 in all calculations.

## 3. Results

This study included 253 women at a mean age of 27.3 ± 4.11 years (the age range of women in the sample was 18–40). The highest percentage of women were aged 25–29 and the majority were urban residents. Nearly 70% of women had a university degree. The percentage of females that underwent vaginal labor was 66.01%, out of whom 31.23% underwent cesarean section (CS) and 2.37% underwent vacuum or forceps delivery. Another 22.11% of females had a vaginal birth (VB) without episiotomy; 41.90% had lateral or posterior episiotomy. The mean wound-healing period was 3.12 ± 2.96 weeks. The mean gestational age was 39 weeks (6.7% of women reported preterm birth, 93.3% had full-term birth). The mean time to resume sexual activity after labor was up to two months. A total of 40.71% of women in the study considered themselves less attractive after childbirth than during pregnancy. The most common contraception method was condoms; 31.62% of women did not use any birth control methods. The majority of respondents were more than three months postpartum. The characteristics of the respondents are presented in Table S1.

Sexual functioning was assessed with the use of the total PL-FSFI score, which varied from 8.4 to 30.0 points, with an average of 20.9 ± 5.59 points. Out of all the PL-FSFI-assessing domains, the highest average score for the whole group was presented for satisfaction (4.12 ± 1.45 points, range: 0–6.0) and the lowest for lubrication (1.62 ± 1.94 points, range: 0–6.0). As many as 58.6% of the participants scored fewer than 27.5 points (FSD). The domain scoring of FSFI for all respondents is presented in Table 1. 

The difference in the changes in PL-FSFI scores and FSD rates between breastfeeding and formula-feeding groups is presented in Table 2.

Of the respondents, 84.71% reported sexual dysfunction in the exclusive breastfeeding group and 78.31% in the formula-feeding group. 

The problems with lubrication and lack of sexual desire were the most prevalent dysfunctions, regardless of the infant feeding method. Significantly lower scores were found in the breastfeeding group compared with the formula-feeding group for the pain subgroup (Pearson Chi^2^: 4.35, df = 1, *p* = 0.04). To assess the effect of the infant feeding method on the index of sexual function, we also used a logistic regression method. We only noted differences for the pain domain. The quality of the model fit is not high (R2Pseudo = 0.013, R2Nagelkerke = 0.023 and R2Cox-Snell = 0.017). At the same time, the model is statistically significant (*p*-value = 0.0348 of the reliability quotient test), so the independent variables in the model are statistically significant. The chance of being diagnosed with FSD in the pain domain depends on the listed variables, as described by the Odds Ratio: breastfeeding: OR [95%CI] = 1.82 [1.03; 3.22] breastfeeding women have a higher chance of being diagnosed with FSD for complaints of pain related to sexuality. The results are presented in the Supplementary Materials (Table S2).

In the other domains (desire, arousal, orgasm, satisfaction), we found no significant differences. Also, there was a non-significant difference: the total FSFI score (Pearson Chi^2^: 1.58638, df = 1, *p* = 0.21), desire (Pearson Chi^2^: 0.935987, df = 1, *p* = 0.33), arousal (Pearson Chi^2^: 3.62750, df = 1, *p* = 0.06), lubrication (Pearson Chi^2^: 0.277847, df = 1, *p* = 0.60), orgasm (Pearson Chi^2^: 0.749812, df = 1, *p* = 0.39), satisfaction (Pearson Chi^2^: 1.73288, df = 1, *p* = 0.19), but in each subgroup there was a central tendency for more cases of FSD in the breastfeeding group.

We noticed significant correlations between time elapsed since childbirth as compared with the pain domain (Pearson Chi^2^: 12.4676, df = 4, *p* = 0.01), and between time elapsed since childbirth as compared with birth option: VB without episiotomy (Pearson Chi^2^: 30.6654, df = 15, *p* = 0.01) and marital status: married (Pearson Chi^2^: 18.8728, df = 10, *p* = 0.04). We also found significantly important correlations in the group which initiated sexual intercourse more often before pregnancy as compared to the recent four weeks (Pearson Chi^2^: 15.3207, df = 2, *p* = 0.00), and we observed that women less often initiated sexual intercourse after childbirth.

In the case of education level, we found a correlation between the arousal domain (Pearson Chi^2^: 7.39960, df = 2, *p* = 0.02) and the satisfaction domain (Pearson Chi^2^: 8.72968, df = 2, *p* = 0.01), and women with higher educational degrees less often had FSD.

In the logistic regression analysis, we showed that the quality of fit of the models is not high (R2Pseudo < 0.04, R2Nagelkerke < 0.06 and R2Cox-Snell < 0.04). 

At the same time, the models are statistically significant (*p*-value < 0.03 of the reliability quotient test), and, therefore, the independent variables in the models are statistically significant. The results of the Hosmer–Lemeshow tests indicate a lack of significance (*p* > 0.1). With the Hosmer–Lemeshow test, we keep in mind that non-significance is desirable because it indicates the similarity of observed counts and predicted probabilities.

The chance of being diagnosed with FSD in the arousal domain depends on the listed variables as described by the Odds Ratio: education: OR [95%CI] = 0.54 [0.34; 0.88]—the higher the participant’s education, the lower the chance of diagnosing FSD at arousal.

The chance of being diagnosed with FSD in the lubrification domain depends on the listed variables as described by the Odds Ratio: time elapsed since completion of pregnancy: OR [95%CI] = 1.37 [1.01; 1.85]—the more time elapsed since completion of pregnancy, the higher the chance of being diagnosed with FSD in the lubrication domain.

The chance of being diagnosed with FSD in the orgasm domain depends on the listed variables as described by the Odds Ratio: time elapsed since pregnancy completion: OR [95%CI] = 0.77 [0.61; 0.97]—the more time elapsed since pregnancy completion, the lower the chance of being diagnosed with FSD in the orgasm domain.

**Figure 2:**
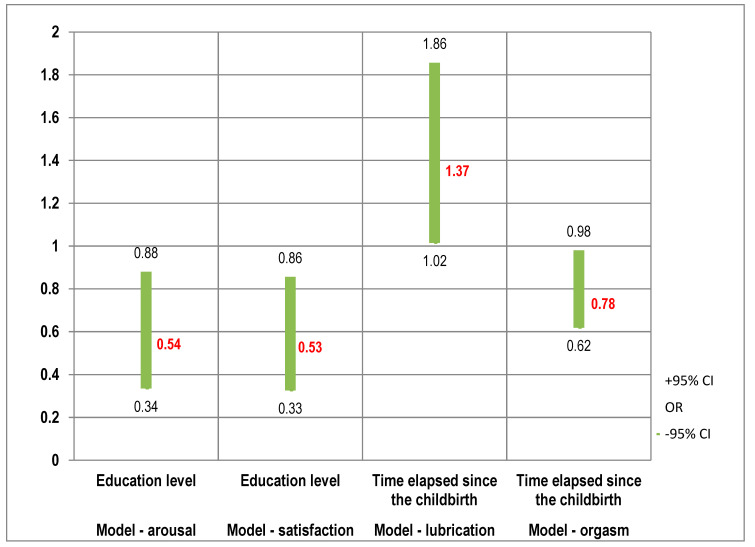
Logistic regression analysis: arousal, lubrication, orgasm and satisfaction domains vs. education level and time elapsed since the childbirth.

The chance of being diagnosed with FSD in the sexual satisfaction domain depends on the listed variables as described by the Odds Ratio: education: OR [95%CI] = 0.52 [0.32; 0.85]—the higher a woman’s education, the lower the chance of being diagnosed with FSD in the sexual satisfaction domain. The results are presented in Figure 2 and Table S3 (Supplementary Materials).

VB without episiotomy as a birth method was also associated with higher satisfaction domain and lower pain domain (Pearson Chi^2^: 13.8253, df = 3, *p* = 0.00 and Pearson Chi^2^: 9.15154, df = 3, *p* = 0.02).

## 4. Discussion

The principal objective of this study was to identify associations between different methods of feeding infants and the sexual functioning of Polish women during the postpartum period.

The most important findings of this study are that regardless of the method of breastfeeding the newborn, women in the postnatal period experience varying degrees of sexual dysfunction, and breastfeeding can be one of the factors that can additionally contribute to the development of certain disorders, due to altered hormonal balance. It seems that in the described population of Polish women, there are other factors related to the socio-economic circumstances of women in Poland which may significantly influence their sexual functioning (such as for instance, where a woman’s lack of child-rearing support contributes to her high levels of fatigue). Such relationships requires further scientific investigation. Additionally, the high rate of perineal incisions among the study group is an important factor: 41.90% had lateral or posterior episiotomy. The wound-healing process and the fact of having undergone an episiotomy can both significantly affect the quality of sexual life. Previous studies indicate similar correlations between sexual dysfunction [25,33,34]. Despite this evidence, few studies have been conducted to identify factors that contribute to the development of FSD among breastfeeding women. Sexual disorders occurring in the breastfeeding period may be related to hyperprolactinemia and physiological hypoestrogenism [20], which can lead to poor vaginal lubrication, increased breast sensitivity, decreased sexual desire and dyspareunia [17,34]. Our study shows that pain is the most prevalent dysfunction in the group of women breastfeeding, which could be connected with the influence of hypoestrogenism on sexual sensation [17,34]. 

According to Yee L. et al. [35] multiparity and younger age can predict early resumption of sexual activity, while breastfeeding and depression are associated with poorer postpartum sexual functioning. In a study conducted by Rådestad et al., breastfeeding was a factor in the delayed resumption of vaginal intercourse after childbirth [31]. Other researchers report similar findings [34,36]. 

Szöllősi K. and Szabó L. [25] indicate that exclusive breastfeeding is associated with an increased likelihood of sexual problems 3 months postpartum. In the breastfeeding group, 50.55% of respondents reported sexual dysfunction, while 42.66% of the mixed feeding group and 31.11% of the formula-feeding group reported sexual dysfunction. In the cited study, the lack of sexual desire was the most prevalent dysfunction (regardless of the feeding method). Moreover, significantly lower median scores were found in the exclusively breastfeeding group compared with the formula-feeding group for the total FSFI score and separate domains who practiced mixed breastfeeding. Only one study suggests no significant difference between women breastfeeding and those formula feeding on women’s sexual desire and satisfaction during the postpartum period [27].

With the increasing amount of scientific evidence that women in the postpartum period experience sexual functioning disorders, authors from various countries observe that counseling in this area is still insufficient. The gestational and postpartum periods provide opportunities to promote positive health behaviors, including health care, mental health, sexual health, and proper nutrition (including the breastfeeding promotion). Moreover, in some countries, especially those with developed health systems, women are more likely to have access to specialized support after childbirth, including sexual counselling. However, even in these countries, the availability and extent of such services may vary. It is important to note that the approach to postpartum care and the availability of sexual counselling may also depend on the culture, traditions and social norms of a country. Sleeping with the infant can be an important factor; it increases the frequency of night feeds and can have a significant impact on the parents’ sexual life [37]. Changes in this area may be occurring in response to a growing understanding of women’s postpartum health needs and increased involvement of the medical community and health organizations. According to researchers, breastfeeding was a risk factor for sexual dysfunction in the arousal and sexual satisfaction domains [38]. Moreover, authors have found that women believed that breastfeeding had a slightly negative influence on the physiologic aspects of sexuality, but did not greatly affect the woman’s sexual relationship with her partner [12]. 

Our study showed that the risk of FSD in the pain domain increased in the group of breastfeeding mothers. An attempt was also made to assess how other factors (such as sociodemographic and obstetric variables) affect the FSFI domains score. We noticed significant correlations between time elapsed since childbirth and pain. These data show that women require the information that some problems can be resolved a few weeks after childbirth.

Importantly, data in the literature indicate that the postpartum period and support for breastfeeding should be complemented by adequate education on sexual function not only for woman, but also for couples. The woman’s partner ought to be integral to the scope of care that addresses changes in the woman’s sexuality during breastfeeding. Supporting the sexual health of women, as well as the protection and promotion of breastfeeding will certainly bring health benefits to the population. 

### 4.1. Limitations

The difference in time elapsed since childbirth for individual respondents may be deemed a limitation of the study, as it may translate into an assessment of individual FSFI domains. Also, it allows for other confounding factors that influence sexual function. The study will be continued with subgroups that include exclusive breastfeeding (up to six months) to examine factors influencing the duration of exclusive breastfeeding closely. Another limitation is the small number of cases, but this preliminary report will allow us to determine power and sample size.

What is more, we acknowledge there is an over-representation of participants with higher education in our group, and this may contribute to an inaccurate characterization of the Polish population; although in recent years, according to the Statistics Poland (GUS), there has been a systematic increase in education levels, especially among women, which makes this group more representative in most survey results [39,40]. Moreover, recent reports show that in terms of education level there has been an adynamic increase of more than 30% for tertiary education in Poland. The practical implication to be drawn from this is that women with higher education are more willing to communicate their problems, which is great, but an additional implication for health professionals is that they need to communicate more deeply with women from groups with different demographic characteristics.

We also recognize the potential for introducing bias through self-reporting and understand the importance of considering additional objective measures in future research. As part of our ongoing efforts, we are exploring the incorporation of complementary objective assessments, such as physiological measures or clinical evaluations, to provide a more comprehensive understanding of sexual functioning after childbirth.

### 4.2. Implications

Given the highest average score correlating with satisfaction and the lowest score correlating with lubrication in our study, it becomes evident that targeted interventions are necessary to address specific aspects of postpartum sexual health. Particularly, our data emphasize the need for healthcare professionals to offer tailored guidance to exclusively breastfeeding women, aimed at preventing and managing sexual problems effectively. In light of our research, we advocate for the integration of postpartum sexual health discussions into routine counseling sessions for couples. Healthcare providers should be equipped with the knowledge and resources to educate couples on the potential of their sexual function, and of their choice of infant feeding methods. This proactive approach is crucial, not only for preserving the sexual health of postpartum women, but also for promoting and sustaining long-term breastfeeding practices.

Recognizing differences in postpartum sexual functioning in the context of breastfeeding will allow for appropriate care planning for postpartum women, given the benefits of prolonged breastfeeding and individual sexual health.

## 5. Conclusions

The most important findings of this work are that regardless of the method of breastfeeding the newborn, women in the postnatal period experience varying degrees of sexual dysfunction, and breastfeeding can be one of the factors that can additionally contribute to the development of certain disorders, due to altered hormonal balance. The differences in feeding methods correlate with the differences in female sexual functions. Our findings indicate that exclusively breastfeeding women have an increased likelihood of sexual problems. Insufficient education in this respect makes women blame themselves, as they feel responsible for losing sexual interest. The lack of sexual interest in the puerperal period, and in fact in the first year of a child’s life, is common and is related to the transition to parenting and the definition of new roles.

This data shows that Polish mothers in the postpartum period need professional support and education about changes in sexual functioning. It is vital to develop strategies for promoting, protecting, and supporting breastfeeding. Promoting the sexual and reproductive health of the breastfeeding woman is recommended in order to clarify possible doubts, include the partner, and prevent the development of disorders, bearing in mind the benefits of breastfeeding and a satisfying sexual life to women’s health. Sexual dysfunctions adversely affect quality of life, self-esteem, and interpersonal relationships. The conducted research proves that sexual disorders need more attention.

## Figures and Tables

**Table 1:** Domain scoring of PL-FSFI.

FSFI Domains	Mean	Median	Min	Max	SD
Desire	3.43	3.60	1.20	6.00	±1.37
Arousal	3.78	4.20	0.00	6.00	±1.79
Lubrication	1.63	1.20	0.00	6.00	±1.94
Orgasm	4.08	4.80	0.40	6.00	±1.72
Satisfaction	4.12	4.40	0.00	6.00	±1.45
Pain	3.91	4.40	0.00	5.60	±1.78
Sexual function	20.95	22.00	8.40	30.00	±5.60

**Table 2:** The difference in the mean total score of PL-FSFI breastfeeding and formula-feeding groups.

FSFI Domains	Formula-Feeding				Breastfeeding			
	FSD		No FSD		FSD		No FSD	
	N	%	N	%	N	%	N	%
**Desire**	48	57.83	35	42.17	109	64.12	61	35.88
**Arousal**	30	36.14	53	63.86	83	48.82	87	51.18
**Lubrication**	71	85.54	12	14.46	141	82.94	29	17.06
**Orgasm**	29	34.94	54	65.06	69	40.59	101	59.41
**Satisfaction**	28	33.73	55	66.27	72	42.35	98	57.65
**Pain**	23	27.71	60	72.29	70	41.18	100	58.82
**Sexual function**	65	78.31	18	21.69	144	84.71	26	15.29

## Data Availability

Data are posted on PPM repository: https://ppm.edu.pl/index.seam (accessed on 8 September 2023).

## Supplementary Materials

The following supporting information can be downloaded at https://www.mdpi.com/article/10.3390/healthcare12010038/s1, Table S1. Respondents characteristics; Table S2: Logistic regression FSFI: pain domain vs. breastfeeding; Table S3: Logistic regression analysis FSF doains vs. time elapsed since the childbirth and educational level.
