# Supplementary material for: Breastfeeding vs. Formula Feeding and Maternal Sexuality among Polish Women: A Preliminary Report
Source: Healthcare (Basel). 2023 Dec 23;12(1):38. doi: 10.3390/healthcare12010038 (PMC10779107; doi:10.3390/healthcare12010038)
Supplement: Supplementary file 1 [file healthcare-12-00038-s001.zip › healthcare-2738326-supplementary.pdf]

**Table S1.** Respondents characteristics.

| Characteristics | Breastfeeding women (n) | % | Formula-feeding Women (n) | % | N | % |
|---|---|---|---|---|---|---|
| *Maternal age [years] MEAN* | | | | | | |
| < 24 | 38 | 22,35 | 27 | 32,53 | 65 | 25,69 |
| 25-29 | 87 | 51,18 | 33 | 39,76 | 120 | 47,43 |
| 30-34 | 35 | 20,59 | 17 | 20,48 | 52 | 20,56 |
| > 35 | 10 | 5,88 | 6 | 7,23 | 16 | 6,32 |
| Place of residence | | | | | | |
| rural area | 33 | 19,41 | 14 | 16,87 | 47 | 18,58 |
| city below 20,000 residents | 10 | 5,88 | 6 | 7,23 | 16 | 6,32 |
| city 20-100 thousand residents | 38 | 22,35 | 22 | 26,50 | 60 | 23,72 |
| city 100-200 thousand residents | 29 | 17,06 | 17 | 20,48 | 46 | 18,18 |
| a city with over 200,000 residents | 60 | 35,30 | 24 | 28,92 | 84 | 33,20 |
| Education | | | | | | |
| Primary school | 5 | 2,94 | 2 | 2,41 | 7 | 2,77 |
| High school | 42 | 24,71 | 28 | 33,73 | 70 | 27,67 |
| University degree | 123 | 72,35 | 53 | 63,86 | 176 | 69,56 |
| Marital status | | | | | | |
| single/divorced | 5 | 2,94 | 3 | 3,62 | 8 | 3,16 |
| married | 134 | 78,82 | 63 | 75,90 | 197 | 77,87 |
| cohabiting | 31 | 18,24 | 17 | 20,48 | 48 | 18,97 |
| Gestational age at delivery [weeks] | | | | | | |
| premature labor (22-27) | 1 | 0,59 | 2 | 2,41 | 3 | 1,19 |
| premature labor (28-31) | 1 | 0,59 | 1 | 1,20 | 2 | 0,79 |
| premature labor (32-36) | 7 | 4,11 | 5 | 6,03 | 12 | 4,74 |
| full-term pregnancy | 161 | 94,71 | 75 | 90,36 | 236 | 93,28 |
| Mode of childbirth | | | | | | |
| Vaginal birth (VB) without episiotomy | 44 | 25,88 | 17 | 20,48 | 61 | 24,11 |
| VB with episiotomy | 70 | 41,18 | 36 | 43,37 | 106 | 41,90 |
| Caesarean section (CS) | 53 | 31,18 | 27 | 32,53 | 80 | 31,62 |
| Vacuum/ forceps delivery | 3 | 1,76 | 3 | 3,62 | 6 | 2,37 |
| Time elapsed since the childbirth | | | | | | |
| up to a month | 8 | 4,71 | 2 | 2,41 | 10 | 3,95 |
| up to three months | 28 | 16,47 | 3 | 3,62 | 31 | 12,26 |
| up to six months | 54 | 31,76 | 17 | 20,48 | 71 | 28,06 |
| to year | 51 | 30,00 | 25 | 30,12 | 76 | 30,04 |
| for a year and a half | 29 | 17,06 | 36 | 43,37 | 65 | 25,69 |
| Who initiated sexual intercourse more often? | | | | | | |
| „my partner” | 111 | 65,29 | 46 | 55,42 | 157 | 62,06 |
| „me” | 59 | 34,71 | 37 | 44,58 | 96 | 37,94 |

| | | | | | | |
|---|---|---|---|---|---|---|
| How do you rate your attractiveness at the moment? | | | | | | |
| “I consider myself more attractive than during pregnancy” | 38 | 22,35 | 19 | 22,89 | 57 | 22,53 |
| “No change” | 63 | 37,06 | 29 | 34,94 | 92 | 36,36 |
| “I consider myself less attractive than during pregnancy” | 69 | 40,59 | 35 | 42,17 | 104 | 41,11 |
| Contraception method | | | | | | |
| natural method (thermal-symptomatic, rhythm method) | 13 | 7,65 | 8 | 9,64 | 21 | 8,30 |
| condom | 67 | 39,40 | 27 | 32,53 | 94 | 37,15 |
| hormone injection | 0 | 0,00 | 1 | 1,20 | 1 | 0,40 |
| OCP (oral contraceptive pill) | 17 | 10,00 | 16 | 19,28 | 33 | 13,04 |
| other methods | 14 | 8,24 | 10 | 12,05 | 24 | 9,49 |
| no contraception methods | 59 | 34,71 | 21 | 25,30 | 80 | 31,62 |
| Time after giving birth to resume sexual activity | | | | | | |
| up to a month | 29 | 17,06 | 13 | 15,66 | 42 | 16,60 |
| up to two months | 90 | 52,94 | 45 | 54,22 | 135 | 53,36 |
| up to three months | 24 | 14,12 | 18 | 21,69 | 42 | 16,60 |
| up to four months | 12 | 7,06 | 4 | 4,82 | 16 | 6,32 |
| to year | 15 | 8,82 | 3 | 3,61 | 18 | 7,12 |

**Table S2. Logistic regression FSFI : pain domain vs breastfeeding**

| **Logistic regression** | |
|---|---|
| **Analyzed variables** | **Pain domain** |
| | **Breastfeeding women** |
| Lack | 0 |
| n | 170 |
| Level of significance | 0,05 |
| Sample size assumption n ≥ 10(k+1) | Tak |
| Sample size assumption n ≥ 10v/p | Tak |
| N | 253 |
| Number of variables in the model | 1 |
| Number of iterations for convergence | 334 |
| The convergence criterion has been achieved | |
| n (no FSD) | 160 |
| n ( FSD) | 93 |
| Reliability quotient test | |
| Log Likelihood | -164,160779 |
| -2 Log Likelihood | 328,321557 |
| Log Likelihood (w. wolny) | -166,387977 |
| -2 Log Likelihood (w. wolny) | 332,775955 |
| Chi-square test | 4,454398 |
| df | 1 |
| p- value | 0,034811 |
| AIC - Akaike's criterion | 330,321557 |
| AICc - adjusted Akaike's criterion | 330,337493 |
| BIC - bayesow's Schwarz criterion | 333,854946 |

| | |
|---|---|
| Pseudo R2 | 0,013386 |
| R2(Nagelkerke) | 0,023855 |
| R2(Coxa-Snella) | 0,017452 |
| Hosmer-Lemeshow test | |
| Chi-square test | NA |
| df | 1 |
| p | NA |

| Model | | | | | | | | | |
|---|---|---|---|---|---|---|---|---|---|
| | b | error b | -95% CI | +95% CI | Wald's statistics | p-value | OR | -95% CI | +95% CI |
| free | -1,561026 | 0,514651 | -2,569723 | -0,552329 | 9,20015 | 0,00242 | 0,209921 | 0,076557 | 0,575608 |
| Breastfeeding women | 0,602175 | 0,29057 | 0,03267 | 1,171681 | 4,29483 | 0,03823 | 1,826087 | 1,033209 | 3,227414 |
| | | | | | | | | | |
| [Hosmer-Lemeshow test] | | | | | | | | | |
| | observed[O] | expected [O] | observed [1] | expected [1] | | | | | |
| 1 | 60 | 16,626506 | 23 | 6,373494 | | | | | |
| 2 | 100 | 41,176471 | 70 | 28,823529 | | | | | |

**Table S3. Logistic regression analysis FSF doains vs time elapsed since the childbirth and educational level**

| Analyzed variables | Arousal domain | | | |
|---|---|---|---|---|
| | Education level | | | |
| Likelihood ratio test | p=0,011766 | | | |
| Pseudo R2 | 0,018243 | | | |
| R2(Nagelkerke) | 0,033154 | | | |
| R2(Coxa-Snella) | 0,02477 | | | |
| Hosmer-Lemeshow test | p=0,261864 | | | |
| Model | p value | OR | -95% CI | +95% CI |
| free | 0,026382 | 7,551067 | 1,267751 | 44,976202 |

| | | | | |
|---|---|---|---|---|
| Education level | 0,013071 | 0,543266 | 0,335541 | 0,87959 |
| | | | | |
| Analyzed variables | Lubrication domain | | | |
| | Time elapsed since the childbirth | | | |
| Likelihood ratio test | p=0,016782 | | | |
| Pseudo R2 | 0,037128 | | | |
| R2(Nagelkerke) | 0,054865 | | | |
| R2(Coxa-Snella) | 0,032045 | | | |
| Hosmer-Lemeshow test | p=0,367514 | | | |
| Model | p value | OR | -95% CI | +95% CI |
| free | 0,031121 | 19,611183 | 1,30997 | 293,5935 |
| Time elapsed since the childbirth | 0,039643 | 1,372945 | 1,015132 | 1,856879 |
| | | | | |
| Analyzed variables | orgazm | | | |
| | Time elapsed since the childbirth | | | |
| Likelihood ratio test | p=0,031535 | | | |
| Pseudo R2 | 0,013769 | | | |
| R2(Nagelkerke) | 0,024745 | | | |
| R2(Coxa-Snella) | 0,018252 | | | |
| Hosmer-Lemeshow test | p=0,291576 | | | |
| Model | p value | OR | -95% CI | +95% CI |
| free | 0,300525 | 1,573057 | 0,667242 | 3,708562 |
| Time elapsed since the childbirth | 0,032785 | 0,778395 | 0,618452 | 0,979702 |
